# Newly Emerging Immune Checkpoints: Promises for Future Cancer Therapy

**DOI:** 10.3390/ijms18122642

**Published:** 2017-12-06

**Authors:** Robert J. Torphy, Richard D. Schulick, Yuwen Zhu

**Affiliations:** Department of Surgery, University of Colorado Anschutz Medical Campus, Aurora, CO 80045, USA; robert.torphy@ucdenver.edu (R.J.T.); richard.schulick@ucdenver.edu (R.D.S.)

**Keywords:** immunotherapy, cosignaling, immune checkpoints

## Abstract

Cancer immunotherapy has been a great breakthrough, with immune checkpoint inhibitors leading the way. Despite the clinical effectiveness of certain immune checkpoint inhibitors, the overall response rate remains low, and the effectiveness of immunotherapies for many tumors has been disappointing. There is substantial interest in looking for additional immune checkpoint molecules that may act as therapeutic targets for cancer. Recent advances during the last decade have identified several novel immune checkpoint targets, including lymphocyte activation gene-3 (LAG-3), B and T lymphocyte attenuator (BTLA), programmed death-1 homolog (PD-1H), T-cell immunoglobulin and immunoreceptor tyrosine-based inhibitory motif domain (TIM-3)/carcinoembryonic antigen cell adhesion molecule 1 (CEACAM1), and the poliovirus receptor (PVR)-like receptors. The investigations into these molecules have generated promising results in preclinical studies. Herein, we will summarize our current progress and understanding of these newly-characterized immune checkpoints and their potential application in cancer immunotherapy.

## 1. Introduction

Targeting cosignaling molecules for cancer immunotherapy is a rapidly expanding area in oncology research. T cell activation after antigen recognition is regulated by an array of cell-surface cosignaling molecules that are either costimulatory or coinhibitory (immune checkpoints) [1]. Key to the understanding of cosignaling molecules is that recognition of an antigen by a T cell is insufficient for T cell activation. This concept was first demonstrated through the costimulatory receptor CD28, which binds to the ligands B7-1 (CD80) and B7-2 (CD86) on antigen-presenting cells (APCs), allowing for T cell activation [2,3]. In contrast, the coinhibitory receptor cytotoxic T-lymphocyte-associated antigen 4 (CTLA-4) acts to prevent excessive immunity, which leads to auto-immune disease, by competing with CD28 for ligand binding, or by directly delivering a negative signal to T cells [4]. With the discovery of varieties of cosignaling molecules, we now understand that every step of T cell-mediated immunity is fine-tuned and counterbalanced by certain groups of costimulatory and coinhibitory signals [5].

**Figure 1:**
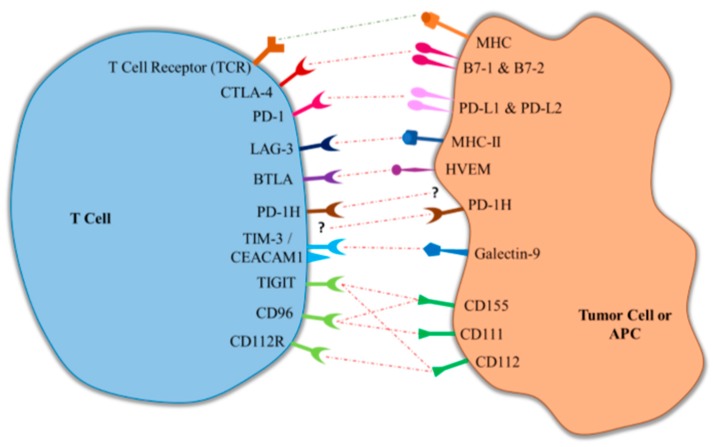
Interaction of immune checkpoint receptors and their respective ligands. LAG-3, lymphocyte activation gene-3; BTLA, B and T lymphocyte attenuator; PD-1H, programmed death-1 homolog; CTLA-4, cytotoxic T-lymphocyte-associated antigen 4; TIM-3, T-cell immunoglobulin- and mucin-domain-containing molecule; TIGIT, T cell immunoglobulin and immunoreceptor tyrosine-based inhibitory motif domain; PD-1, programmed death protein-1; CEACAM1, carcinoembryonic antigen cell adhesion molecule 1; MHC-II, class II major histocompatibility complex; FDA, United States Food and Drug Administration; APCs, antigen-presenting cells; HVEM, herpesvirus entry mediator.

Targeting cosignaling molecules to modulate immune responses holds great promise for cancer immunotherapy. In numerous pre-clinical studies, amplifying costimulatory molecules has been demonstrated to be an effective method in treating tumors. Although temporarily hampered by severe side effects in a phase I clinical trial of a super-agonist for CD28 [6], many agents targeting costimulatory molecules, including at least 4-1BB, OX-40, CD40, and ICOS, are currently under clinical testing for cancer therapy [7,8,9]. On the other hand, the immune response against tumors can be augmented by blocking immune checkpoints. Supporting that, the expression of immune checkpoints has been shown to be altered in certain tumors disrupting the antitumor immune response [1]. Breakthroughs have subsequently been made in clinical cancer immunotherapy by targeting checkpoints CTLA-4, and especially programmed cell death protein 1 (PD-1), which has resulted in United States Food and Drug Administration (FDA) approval for anti-CTLA-4 and anti-PD-1 therapies. Despite these advancements, significant gaps exist in cancer immunotherapy, as only a subset of cancers have demonstrated clinical response to CTLA-4 and PD-1 targeted therapies [10]. In this review, we will briefly discuss the progress to date in cancer therapy related to immune checkpoint inhibitors, and focus on describing newly-emerging immune checkpoints under active clinical development, and their potential for future cancer therapy (Table 1 and Figure 1).

## 2. Progress in Cancer Immunotherapy

### 2.1. Cytotoxic T-Lymphocyte-Associated Antigen 4 (CTLA-4)

CTLA-4 was first identified as a homologue of CD28, which is expressed on activated CD4+ and CD8+ T cells and binds the same ligands as CD28, B7-1 and B7-2 [11,12]. CTLA-4 was subsequently shown to be an inhibitory receptor on T cells, as blockade of CTLA-4 enhanced antitumor immunity and CTLA-4 knockout mice developed severe lymphoproliferative disease [13,14].

James Alison’s group was the first to demonstrate that the blockade of the CTLA-4 signal by a monoclonal antibody (mAb) alone can cause tumor regression in a transplanted mouse tumor model [15]. The susceptibility of tumors to regress with CTLA-4 blockade was further shown to correlate with the immunogenicity of the tumor, and the response in poorly immunogenic tumors could be augmented by co-treatment with granulocyte-macrophage colony-stimulating factor (GM-CSF) vaccines [16,17]. In addition to inhibiting the CTLA-4 inhibitory signal, recent work has suggested that selectively depleting regulatory T (Treg) cells at tumor sites, which happen to express the highest level of surface CTLA-4, is critical for CTLA-4 mAb-mediated antitumor effect [18].

In 2010, ipilimumab, a human monoclonal antibody that blocks CTLA-4, was demonstrated to improve overall survival in patients with metastatic melanoma [19]. Importantly, a subset (~10%) of patients experienced a long-term survival benefit. Subsequently, ipilimumab was approved in the United States and Europe for the treatment of unresectable or metastatic melanoma in 2011, becoming the first immune checkpoint to be clinically targeted in cancer immunotherapy. Despite the survival benefits of ipilimumab in metastatic melanoma, significant side effects of anti-CTLA-4 treatment include the associated immune-related adverse events (60% in patients treated with ipilimumab) which most commonly affect the gastrointestinal tract [19]. The high incidence of immune-related adverse events with anti-CTLA-4 treatment is likely due to the depletion of Treg cells and systemic activation of autoreactive T cells in lymphoid tissue [20].

### 2.2. Programmed Death Protein 1 (PD-1)/Protein Death-Ligand 1 (PD-L1)

PD-1 was the second immune checkpoint to come to the forefront of cancer immunotherapy. PD-1 was initially cloned from B cell lymphoma, and was demonstrated to be upregulated when T cells are activated [21]. The known ligands of PD-1 are PD-L1 (B7-H1) and PD-L2 (B7-DC) and upon binding these ligands, PD-1 acts to inhibit T cells [22]. Mice deficient in PD-1 demonstrate auto-immune disease, albeit less than CTLA-4-deficient mice, including a lupus-like syndrome and cardiomyopathy, further demonstrating the inhibitory role of PD-1 in autoimmunity [23,24]. One promising attribute of PD-1 in cancer immunotherapy is that the expression of PD-1 ligands, especially PD-L1, has been shown to be upregulated in a variety of tumors, and blockade of PD-L1 signal can sensitize tumors to cytotoxic T lymphocyte (CTL) killing [25]. These data suggest that anti-PD-1 treatments may be more specific in targeting cancer cells, resulting in less systemic toxicity.

In the first clinical trial of a PD-1 mAb for cancer patients conducted by The Johns Hopkins Hospital, objective response was observed in multiple cancer types, including melanoma, renal cell cancer, and non-small-cell lung cancer. The toxicity associated with drug effect was mild, and the anticancer effect was durable [26]. Pembrolizumab and nivolumab are commercially available humanized monoclonal antibodies against PD-1 that have demonstrated a wider breadth of clinical utility than iplimumab. Pembrolizumab treatment has resulted in survival benefits in patients with melanoma, non-small-cell lung cancer, advanced urothelial carcinoma, squamous cell carcinoma of the head and neck, and gastric cancer, and has received approval for the treatment of select patients with these malignancies [27,28,29,30]. Phase III studies for a variety of malignancies and select patient populations are still underway. Nivolumab has also demonstrated efficacy in melanoma, non-small-cell lung cancer, urothelial carcinoma, squamous cell carcinoma of the head and neck, renal cell cancer, hepatocellular cancer, and metastatic colorectal cancer [31,32,33,34,35,36,37]. Importantly, in trials comparing PD-1-directed therapy vs CTLA-4-directed therapy, patients receiving anti-PD-1 treatment demonstrate fewer immune-related adverse events.

Anti-PD-L1 therapy is another approach for disrupting PD-1/PD-L1 signaling. PD-L1 and PD-1 have been demonstrated to be expressed, respectively, by human cancer cells and tumor-infiltrating lymphocytes (TILs). PD-L1 expression on the surface of tumor cells can be upregulated by interferon-γ produced by TILs, suggesting that this is an adaptive mechanism of tumor cells [38]. Upregulation of surface PD-L1 on tumor cells increases apoptosis of tumor-reactive T cells and promotes tumor growth in mouse tumor models [10,39]. Atezolizumab is the first anti-PD-L1 mAb that received accelerated approval in the United States for the treatment of metastatic urothelial carcinoma after promising phase I/II results [40]. Disappointingly, in a subsequent phase III trial, there was no overall survival benefit from treatment with atezolizumab vs chemotherapy in advanced bladder cancer [41]. Atezolizumab is also approved for the treatment of non-small-cell lung cancer, where it has demonstrated a survival advantage in phase II and III clinical trials [42,43]. Durvalumab and avelumab are the other two anti-PD-L1 mAbs approved for patients with locally advanced or metastatic urothelial carcinoma that fails platinum-containing chemotherapy [44]. All these drugs targeting PD-L1 are under active clinical trials for multiple solid cancer types.

## 3. Newly Emerging Immune Checkpoints

### 3.1. Lymphocyte Activation Gene-3 (LAG-3)

Lymphocyte activation gene-3 (LAG-3 or CD223), a single transmembrane protein with three-Ig extracellular domain, is expressed on activated T cells, regulatory T cells, natural killer (NK) cells, dendritic cells (DCs), and B cells [45,46]. Class II major histocompatibility complex (MHC-II) has been identified as a binding ligand for LAG-3, an interaction that was identified secondary to the structural homology between LAG-3 and CD4 [47]. Evidence to date suggests LAG-3 acts as a coinhibitory molecule. Blocking LAG-3 with a mAb resulted in more T cell proliferation in vitro; and in a LAG-3 knockout mouse, this receptor regulated the homeostatic expansion of CD4 and CD8 T cells [48]. LAG-3 is co-expressed with other immune checkpoints in exhausted T cells, and blockade of LAG-3 on exhausted CD8+ T cells resulted in restoration of immune function, which was synergistic with concurrent PD-1 blockade [49]. LAG-3 was also found to be upregulated on Treg cells, and conferred regulatory function. Blocking LAG-3 on Treg cells both in vitro and in vivo resulted in reduction of suppressor activity [45]. These findings suggest that LAG-3 acts to inhibit the immune response by directly inhibiting effector T cell killing, and through Treg cell-mediated immune suppression.

Soluble LAG-3 has been commercially developed for further clinical testing given the promising pre-clinical findings and potential in augmenting the anti-tumor immune response. In addition to blocking the negative LAG-3 signal, soluble recombinant LAG-3-Fc fusion protein (LAG-3-Ig) augments the immune response by inducing DC activation [50]. This process of DC activation is also dependent on MHC-II binding [47]. In the presence of LAG-3-Ig, DCs have an increased capacity to induce T cell proliferation and interferon-γ production [51]. This role in activating DCs has been further demonstrated in mouse models, where LAG-3-Ig used as a vaccine adjuvant has been shown to improve the anti-tumor immune response [52,53]. IMP321 was the first commercially developed soluble LAG-3 protein, and is a LAG-3 dimer. Multiple phase I and phase II trials have been completed, with IMP321 as a vaccine adjuvant (influenza, hepatitis, melanoma, prostate cancer), in combination with chemotherapy (breast cancer, pancreatic cancer), and as a single agent (renal cell carcinoma) [54,55,56,57,58]. These early-phase trials have shown a mild side effect profile, and promising early results, suggesting IMP321 may enhance T cell response to antigen vaccination. An ongoing phase I clinical trial is investigating IMP321 in combination with pembrolizumab in melanoma (clinicaltrials.gov, NCT02676869), and an ongoing phase I clinical trial is investigating IMP321 in combination with chemotherapy in patients with metastatic breast cancer (clinicaltrials.gov, NCT02614833). LAG525 is an antagonist LAG-3 mAb that is in phase I study both as a single agent and a combination treatment with a PD-1 inhibitor for solid tumors (clinicaltrials.gov, NCT02460224).

### 3.2. B and T Lymphocyte Attenuator (BTLA)

B and T lymphocyte attenuator (BTLA) is an immunoglobulin domain-containing glycoprotein expressed on T cells, resting B cells, macrophages, DCs and, to a lesser extent, NK cells [59,60]. BTLA acts as an inhibitory receptor on T cells, as anti-BTLA treatment results in T cell proliferation, and BTLA knockout mice demonstrate hyper-responsive immune activation [59]. Subsequently, herpesvirus entry mediator (HVEM), a tumor necrosis factor receptor, was identified as a natural ligand for BTLA in mice and humans. Expression of HVEM by antigen-presenting cells (APCs) was capable of inducing BTLA-dependent T cell inhibition [60].

BTLA belongs to the immunoglobulin superfamily, along with CTLA-4 and PD-1, which characteristically binds to B7 family members. HVEM is a member of the tumor necrosis factor receptor family, and the BTLA/HVEM interaction is the first to demonstrate crosstalk between these two receptor families. Prior to the discovery of the BTLA/HVEM interaction, HVEM was known to bind lymphotoxin-α and LIGHT, both tumor necrosis factor ligands. While the BTLA/HVEM interaction results in a co-inhibitory signal, the HVEM/LIGHT interaction results in a co-stimulatory signal through HVEM [61].

In melanoma patients, BTLA was demonstrated to be expressed on tumor-specific T cells both in circulation and in metastatic lymph nodes. HVEM was subsequently shown to be expressed in the majority of cultured melanoma cell lines and metastatic melanoma samples. The interaction of BTLA on tumor specific T cells and HVEM on melanoma cells resulted in T cell inhibition, which could be reversed by treatment with a BTLA blocking mAb [62]. Therapeutically targeting BTLA and HVEM remains in pre-clinical stages as this bidirectional signaling pathways of BTLA/HVEM and HVEM/LIGHT are further elucidated.

### 3.3. Programmed Death-1 Homolog (PD-1H)

Programmed death-1 homolog (PD-1H) is a cell surface molecule that was discovered secondary to its immunoglobulin variable domain homology with PD-1 [63]. PD-1H is also referred to as V-domain Ig suppressor of T cell activation (VISTA) as it was initially shown to inhibit T cell activation [64]. While PD-1H has homology to PD-1, PD-L1 and PD-L2, as well as other members of the B7 family, there are distinct differences in the expression pattern of PD-1H. PD-1H expression is present in hematopoietic cells, particularly APCs as well as CD4+ lymphocytes [64,65]. PD-1H expression on APCs has been shown to inhibit both CD4+ and CD8+ T cells, and blockade of PD-1H was shown to potentiate the development of T cell mediated autoimmunity [64]. The inhibitory role of PD-1H on APCs acts independently of the PD-1 receptor in T cells, suggesting that PD-1H on APCs functions as a coinhibitory ligand and binds to a yet-to-be-identified T cell receptor. PD-1H is also found constitutively expressed on naïve CD4+ T cells, where it was shown to act as a coinhibitory receptor. PD-1H knockout mice are susceptible to autoimmune disease induction [66]. Inconsistent with that, PD-1H knockout mice are resistant to glioma challenge in a CD4+ T cell-mediated mechanism [65]. In combination, these studies suggest that PD-1H can act to inhibit T cells as both a ligand and a receptor.

PD-1H immunotherapy is in pre-clinical and clinical studies. JNJ-61610588 is a humanized IgG1 Kappa anti-VISTA antibody currently in phase I trials for advanced solid malignancies (clinicaltrials.gov, NCT02671955).

### 3.4. T-Cell Immunoglobulin- and Mucin-Domain-Containing Molecule (TIM-3)/Carcinoembryonic Antigen Cell Adhesion Molecule (CEACAM1)

T-cell immunoglobulin- and mucin-domain-containing molecule (TIM-3) was originally discovered in 2002 as a Th1 specific cell surface protein not expressed on naïve T cells, B cells, macrophages or DCs [67]. TIM-3 is an activation-induced inhibitory molecule that has been shown to bind the ligand galectin-9 [68]. Furthermore, TIM-3 has been implicated in tolerance, and shown to induce T-cell exhaustion in chronic viral infections and cancer [69]. In cancer patients, TIM-3 was found on CD8+ T cells along with PD-1 resulting in a dysfunctional T cell phenotype. Blockade of TIM-3 alone or in conjunction with PD-1 blockade reversed this T cell dysfunction [69].

Carcinoembryonic antigen cell adhesion molecule 1 (CEACAM1) is also expressed on activated T cells, and has been shown to interact with TIM-3, an interaction that is required for TIM-3-mediated T cell inhibition [70]. CEACAM1 has been studied extensively in the immune system and cancer, where it was shown to be expressed on T cells, NK cells, and a variety of tumor cells [71]. Prior to identifying the TIM-3/CEACAM1 interaction, CEACAM1 was also independently identified as an immune checkpoint receptor expressed on activated T cells [72]. CEACAM1 is suggested to have both coinhibitory and costimulatory functions, depending on which CEACAM1 isoform is involved. CEACAM1-L is the dominant isoform expressed on T cells, and has a coinhibitory function, while CEACAM-S, a less common isoform, has a costimulatory function [71].

Pre-clinical studies targeting TIM-3 and CEACAM1 as cancer immunotherapy targets are promising. In mice, TIM-3 and CEACAM1 monoclonal antibody treatment resulted in a reduced tumor burden when administered independently, and demonstrated an additive benefit when administered together. This treatment was also associated with increased T cell activation [70]. Two anti-TIM-3 mAbs (TSR-022 and MBG453) are currently in phase I clinical trials for advanced solid tumors (clinicaltrials.gov, NCT02817633 and NCT02608268).

### 3.5. Poliovirus Receptor (PVR)-Like Proteins (TIGIT, CD96, CD112R)

Poliovirus receptor (PVR)-like receptors are an emerging group of T cell receptors with immunomodulatory functions [73]. CD226 and T cell immunoglobulin and immunoreceptor tyrosine-based inhibitory motif domain (TIGIT) are two PVR-like receptors implicated in immunomodulation through their interaction with the ligands CD155 and CD112. CD226 interacts with CD155 and CD112 to costimulate T cells, while TIGIT interacts with these two ligands and inhibits T cell response [74]. CD155 likely acts as the dominant ligand in this ligand/receptor network, because the interaction between CD112 and TIGIT is weak [74,75].

TIGIT has been shown to be highly expressed, in correlation with PD-1 expression, in TILs of multiple cancer types. In mouse tumor transplant models, treatment with TIGIT-blocking antibodies alone did not decrease tumor burden, but treatment with TIGIT blocking antibodies in combination with anti-PD-L1 or anti-PD-1 treatment brought about a robust anti-tumor response [76]. The synergism of TIGIT blockade in combination with PD-1 or PD-L1 blockade was further demonstrated in T cells from patients with advanced melanoma, where TIGIT was also found to be co-expressed with PD-1. The TIGIT ligands (CD155 and CD112) were also shown to be upregulating in melanoma cells and APCs from melanoma patients [77,78]. OMP-313M32 is an anti-TIGIT monoclonal antibody that is currently in Phase I clinical trial (clinicaltrials.gov, NCT03119428).

CD96, initially named Tactile (T cell activation, increased late expression), is another PVR-like co-receptor present on T cells and NK cells [79]. CD96 interacts with CD155 and CD111, but not CD112. CD96 competes with CD226 for CD155 binding and acts to negatively regulate NK cells [80,81]. Antibody blockade of CD96 in mouse models promoted NK cell function and anti-tumor response, both alone and in combination with anti-CTLA-4 or anti-PD-1 treatment [82]. CD112R, also named PVRIG, is a recently identified surface protein that is also a member of the PVR-like protein family [83]. CD112R interacts with CD112 but not CD155, and appears to compete with CD226 for CD112 binding. Blocking the CD112R/CD112 interaction with a CD112R blocking antibody enhanced T cell stimulation, indicating that CD112R acts as a coinhibitory receptor [83]. Blockade of CD112R, as well as TIGIT on human NK cells, enhanced trastuzumab-mediated NK cell anti-breast-cancer response, further demonstrating the coinhibitory role of CD112R [84].

## 4. Future Outlook

The future of cancer immunotherapy is very promising, and combination checkpoint targeting treatments will likely lead the way in improving clinical response rates. We now know that different immune checkpoints regulate different stages and cell types of the immune response. Anti-CTLA-4 and anti-PD-1 combination therapy has already demonstrated improved clinical response rates in melanoma compared to either agent alone [85,86]. CTLA-4 is thought to play a role predominantly in regulating the magnitude of early T cell activation, while PD-1 is expressed upon T cell activation and regulates effector T cell activity. Furthermore, CTLA-4 expression is restricted to only T cells, while PD-1 is found on T cells, B cells and NK cells. By simultaneously blocking CTLA-4 and PD-1, clinical anti-tumor response should improve, as their inhibitory signals will be blocked at multiple steps and in multiple cell types. An understanding of the newly-emerging immune checkpoints described above will open the door to different combination therapies that may better modulate the immune system to promote an anti-tumor response and limit auto-immune related side effects.

These newly emerging immune checkpoints may also be used in combination with other immune therapies to help augment the immune response. We know that only a subset of cancers currently show favorable response rates to checkpoint immunotherapy, in part due to varying immunogenicity of different tumors and immunosuppressive tumor microenvironments. Pancreatic cancer is an example of a highly lethal cancer that has been shown to have a dearth of tumor-infiltrating effector T cells, and which has failed to respond to single-agent checkpoint inhibitors [87,88]. Vaccine immunotherapy offers the possibility of priming the immune system to tumor antigens, allowing for a more robust response to checkpoint inhibitors. With the improvement of DNA sequencing technology, the combination of immune checkpoint inhibitors with neoantigen vaccines could offer a new approach for optimizing anti-tumor T cell immunity [89].

In summary, numerous immune checkpoints have been identified to date, and are in varying stages of pre-clinical and clinical development (Table 1). Combination immune checkpoint therapy and combination anti-tumor vaccine therapy with immune checkpoint therapy are both promising methods to improve response rates and broaden the use of immunotherapy across different tumor types.

## Figures and Tables

**Table 1:** Summary of co-inhibitory immune checkpoint receptors.

Co-Inhibitory Checkpoint	Receptor Expression	Binding Partner	Binding Partner Expression	Clinical Development
CTLA-4	Effector T cells Regulatory T cells	B7-1 (CD80) B7-2 (CD86)	APCs Tumor MDSCs	FDA Approved (ipilimumab)
PD-1	TILs Effector T cells Regulatory T cells B cells NK cells	PD-L1 (B7-H1) PD-L2 (B7-DC)	Cancer cells APCs Tumor MDSCs	FDA Approved (pembrolizumab and nivolumab)
LAG-3	Effector T cells Regulatory T cells B cells NK cells Dendritic Cells	MHC Class II	APCs	Phase I/II Clinical Trials (IMP321 & LAG525)
BTLA	T cells B cells NK cells	HVEM	Cancer cells APCs T cells	Pre-Clinical Studies
PD-1H	T cells APCs	Unknown	N/A	Pre-Clinical Studies Phase I Clinical Trials (JNJ-61610588)
TIM-3/CEACAM1	Effector T cells	Galectin-9	Cancer cells APCs	Pre-Clinical Studies Phase I Clinical Trials (TSR-022 & MBG453)
TIGIT	Effector T cells Regulatory T cells NK cells	CD155 CD112	Cancer cells APCs	Pre-Clinical Studies Phase I Clinical Trial (OMP-313M32)
CD96	Effector T cells Regulatory T cells NK cells	CD155 CD111	Cancer cells APCs	Pre-Clinical Studies
CD112R	Effector T cells NK cells	CD112	Cancer cells APCs	Pre-Clinical Studies

LAG-3, lymphocyte activation gene-3; BTLA, B and T lymphocyte attenuator; PD-1H, programmed death-1 homolog; CTLA-4, cytotoxic T-lymphocyte-associated antigen 4; TIM-3, T-cell immunoglobulin; TIGIT, T cell immunoglobulin and immunoreceptor tyrosine-based inhibitory motif domain; PD-1, programmed death protein-1; CEACAM1, carcinoembryonic antigen cell adhesion molecule 1; MHC-II, class II major histocompatibility complex; FDA, United States Food and Drug Administration; APCs, antigen-presenting cells; MDSCs, myeloid derived suppressor celsl, HVEM, herpesvirus entry mediator.
